# Sampling plan of SB Brasil 2023: precision of dmft and DMFT estimates for the study domains

**DOI:** 10.1590/1807-3107bor-2025.vol39.044

**Published:** 2025-05-19

**Authors:** Maria Cecília Goi Porto ALVES, Gizelton Pereira ALENCAR, Andrea Maria Duarte VARGAS, Raquel Conceição FERREIRA, Regina Tomie Ivata BERNAL

**Affiliations:** (a) Secretaria de Estado da Saúde de São Paulo, Health Institute, São Paulo, SP, Brazil.; (b) Universidade de São Paulo – USP, School of Public Health, Department of Epidemiology, São Paulo, SP, Brazil.; (c) Universidade Federal de Minas Gerais – UFMG, School of Dentistry, Departament of Social and Preventive Dentistry, Belo Horizonte, MG, Brazil.; (d) Universidade Federal de Minas Gerais – UFMG, Nursing School, Belo Horizonte, MG, Brazil.

**Keywords:** Epidemiology, Health Surveys, Dental Health Surveys, Cluster Sampling, Data Accuracy

## Abstract

The oral health surveys conducted in Brazil since the 1980s, aligned with the guidelines of the National Oral Health Policy, have been essential for epidemiological surveillance. Over the surveys, variations in the applied sampling plans have occurred, including changes in the study domains. In SB Brasil 2023, an effort was made to meet the demands of state managers by expanding the domains including Federative Units and capitals. This study presents the sampling plan and assesses the precision of dmft and DMFT estimates for the defined domains. The sampling process was stratified (capitals and interior of the Federative Units) and involved a two-stage cluster design (census tract and households) for the age groups 15–19, 35–44, and 65–74 years, while a single-stage design was used for the ages of 5 and 12 years. The planned sample size was 250 (for ages 5 and 12) or 300 (for the other age groups) in the capitals, with an additional 100 interviews in the interior to obtain estimates for the Federative Units. The number of census tracts in each stratum was determined to achieve 250 interviews for the ages of 5 or 12 years. During the data analysis phase, base weights were adjusted through post-stratification based on sex, age, and education level, using data from the 2022 Continuous National Household Sample Survey, aiming to minimize selection and response biases. The dmft and DMFT estimates were evaluated using the coefficient of variation. Most estimates were precise, both for the capitals and for the Federative Units, with greater precision in the capitals.

## Introduction

The sampling plan is a crucial step in designing population surveys. Its development involves decision-making based on criteria of precision and validity of the indicators to be obtained, without neglecting the feasibility aspects of the chosen process.^1^ This planning, guided by the survey objectives, should lead to data collection capable of informing the evaluation of policies, programs, and health decisions.^1^

In Brazil, within the area of oral health, population-based surveys have emerged as a key strategy for obtaining primary data for oral health surveillance actions. This strategy aligns with the guidelines of the Brazilian National Oral Health Policy, which guides the application of epidemiological information on the health and disease conditions of the population for planning oral health actions.^2,3^ Before SB Brasil 2023, four major oral health epidemiological surveys have already been conducted (1986, 1996, 2003, and 2010). However, it was from 2003 on that the sampling plans sought estimates for the population in age groups defined by the World Health Organization to assess oral health in children (5 years), adolescents (12 and 15–19 years), adults (35–44 years), and elderly people (65–74 years).^4-6^

The geographical domains to be considered in the surveys have also changed across editions.^4,6,7^ SB Brasil 2023 expanded the number of domains to meet the needs of oral health service management in the Brazilian Unified Health System (SUS).^8^ In addition to the 26 capitals adopted in 2010 as study domains, all 27 Federative Units were also considered. The goal was to preserve the ability to obtain estimates for capital cities for historical analyses of oral health issues while also considering the demands of state managers for data to support decision-making at this level. As a result, it was necessary to develop a viable sampling plan that would allow for the collection of estimates for each Federative Unit, considering both capital cities and interior municipalities in the sample composition. This decision led to an increase in the number of required interviews and examinations, and a less concentrated distribution of the sample across the country compared to 2010, presenting significant challenges for obtaining precise estimates for all domains.

This study aims to present the sampling plan used in SB Brasil 2023 and evaluate the precision of dmft and DMFT estimates for the defined study domains. The registration and detailed description of the sampling plan, in addition to providing transparency to the process, guide the interpretation and use of the data, and also serve as an experience for future national surveys.

## Methods

### Sampling plan

The study population consisted of Brazilians residing in permanent private households, in the urban areas of the entire national territory, in 2023. The study domains considered were the 27 Federative Units (26 states and the Federal District) and the capitals, totaling 53 geographical domains. The population groups in the ages of 5 and 12 years, as well as in the age groups of 15–19, 35–44, and 65–74 years, comprised the demographic domains.

The population was divided into 53 strata: the Federal District, capitals, and interior municipalities of each Federative Unit. The population residing in these strata was estimated by applying the population data from the 2010 census, with the percentage changes between 2010 and 2019 by age, proposed by the Brazilian Institute of Geography and Statistics (IBGE) for the Federative Units.^9^ The same percentages were used for both capital cities and the interior of the Federative Units. Regarding households and census tracts, 2019 data, prepared by IBGE in anticipation of the 2020 census,^10^ were considered. Census tracts with fewer than 20 households were excluded, representing 0.08% of the study population, along with the corresponding households. Census tracts with 500 or more households were divided as follows: those with 500 to 799 households were split into two parts; those with 800 to 1199 were divided into three parts; and those with 1200 or more households were split into four parts.

The capitals’ sample size was defined as 250 for the ages of 5 and 12 years and 300 for the other age groups (15–19, 35–44, and 65–74 years). Based on the algebraic expressions for calculating the necessary sample size to estimate the means, $n=\frac{s_{y}^{2}}{(d/z{)}^{2}}\cdot deff$ , and for proportions, $n=\frac{p\cdot (1-p)}{(d/z{)}^{2}}\cdot deff$ , the sampling errors (d) associated with estimates *ȳ* (mean of dmft or DMFT) and p (proportions of malocclusion, bleeding, calculus presence, pocket presence, use of upper and lower dentures, need for upper and lower dentures and trauma) were examined. Using the estimates obtained in SB Brasil 2010 for s_*y*_ (standard deviation of y) and p for all capitals; considering *deff*=2 (design effect) and z=1.96 (95% confidence level for confidence intervals), it was found that the proposed sample sizes would be sufficient to estimate means of dmft or DMFT with sampling errors corresponding to less than one tooth for ages 5, 12, and 15–19 years, and less than 1.3 teeth for the age groups 35–44 and 65–74 years. The estimates for proportions would be obtained with sampling errors smaller than 9% for the index ages of 5 and 12 years, and smaller than 8% for the other age groups. It was further defined that 100 interviews would be conducted in the interior of the Federative Units so that the sample size would be 350 for the ages of 5 and 12 years and 400 for the other age groups. This addition was justified by the expectation of a higher design effect in the estimates obtained for the combined set of interviews, both capital and interior, in each Federative Unit.

To calculate the number of households needed to obtain the planned examinations, it was assumed that 40% of the selected individuals would not participate, due to vacant households, households with no resident contact to check for eligible individuals (closed or refusal to provide information), and eligible individuals who would not participate in the survey (absence during the visits by examiners and refusal). Thus, the calculation of the number of households in the sample was made considering samples of 420 people (for 5 and 12 years) and 500 people (for the other age groups) in the capitals, and 170 people in the interior.

A stratified cluster sample, was randomly selected in one or two stages. For the ages of 5 and 12, the sample was obtained in a single stage, by searching for children and adolescents of these ages in all households within the selected census tracts. The sample was obtained in two stages for the other age groups: census tract and household.

The number of census tracts in each stratum was defined by dividing the sample size by the ratio between 12-year-old children and households, since for this group, as well as for 5-year-olds, all households in the census tract would be surveyed. Thus, it was decided to accept that the sample for 5-year-old children would be slightly smaller than planned when the number of 5-year-olds was lower than the number estimated for 12-year-olds. In the interior, 458 census tracts located in 395 distinct municipalities were drawn, and 1,365 census tracts were selected in the capitals. In each stratum, reserve census tracts were drawn, totaling 20% of the number of census tracts in the sample.

The selection of sampling units was done using probability proportional to size, given by the number of permanent private households. In each stratum, the corresponding sampling fraction for this sampling process is: $f=\frac{a\cdot M_{i}}{M}\cdot \frac{b}{M_{i}^{\prime }}=\frac{a\cdot b}{M}$ , where a is the number of census tracts to be selected, b is the number of households to be selected in each census tract, Mi is the number of households in census tract i, according to the 2019 version of IBGE’s data for the preparation of the Census 2022, and M is the total number of households in the stratum. If the number of households found during fieldwork in the selected census tracts differed from the census data, the values of b would remain, and thus the sampling fraction would be adjusted to: $f=\frac{a\cdot M_{i}}{M}\cdot \frac{b}{M_{i}^{\prime }}=\frac{a\cdot b}{M}\cdot \frac{M_{i}}{M_{i}^{\prime }}$ , where M_*i*_’ is the current number of households For the index ages of 5 and 12 years, where all children residing in the census tracts would be included in the sample (b=M_i_), $f=\frac{a\cdot M_{i}}{M}\cdot \frac{M_{i}^{\prime }}{M_{i}^{\prime }}=\frac{a\cdot M_{i}}{M}$ .

Difficulties encountered during fieldwork led to changes in the proposed sampling fractions. After a few months of fieldwork, a high non-response rate was observed in the phase of identifying the eligible population for many of the census tracts already visited. This led to a significant reduction in the number of households available for the planned examinations. The sampling fraction for census tracts not yet visited was then adjusted so that more households would be visited, with a subsequent random selection of those with eligible residents in the age groups of interest. Later, in the strata where it was assessed that this measure would be insufficient to achieve the sample size, another change was introduced in census tracts that had not yet worked. It was then decided to visit all households to search for the eligible population. These changes resulted in the sampling fraction being rewritten as $f=\frac{a\cdot M_{i}}{M}\cdot \frac{b_{i}^{\prime }}{M_{i}^{\prime }}$ , where $b_{i}^{\prime }=b\cdot \frac{t_{i}}{T_{i}}$ , t_*i*_ is the number of households where the eligible population was found, and T_i_ is the number of households visited in census tract i. The census tracts in the same stratum now had different fractions depending on the selection alternative used, but the expression above covers all of them. The initial sampling fraction, where t_i_ = T_i_, is a special case of this fraction now described. The census tracts were worked on using a single selection method, as the changes were always applied to census tracts where the household selection had not yet been carried out.

The inverse of the updated sampling fractions constituted the base weight. To minimize selection and response bias in the survey, these weights were adjusted using post-stratification weights through the Rake method. The goal was to equalize the joint distributions of sex, age, and education for the sample and reference population. Population data were extracted from the Continuous National Household Sample Survey (PNAD) for the fourth quarter of 2022,^11^ and weights were calculated using the Stata® program with the SURVWGT package.^12^ Missing data in the sample for sex and education level by Federative Unit and age group, necessary for post-stratification adjustment procedures, were imputed using the Decision Tree technique through the “rpart” package in R.^13^ The “weight trimming procedures”^14,15^ were also applied to trim outliers in the post-stratification weight distribution. The trimmed weights were calculated using the Calculate Sample Weight module of the SISA program, provided by Quantitative Skill.

### Evaluation of the plan

The epidemiological indicators selected for assessing the sampling plan were the indices of missing, decayed, and filled teeth, both for deciduous dentition at age 5 (dmft) and for permanent dentition in other age groups (DMFT). This index is recommended by the WHO for assessing caries experience,^16^ one of the most prevalent oral diseases in the population.

To evaluate the precision of the dmft/DMFT estimates, the coefficient of variation was used. The coefficient of variation is the most commonly used measure for assessing survey results.^17^ It was calculated as the ratio between the standard error of the dmft/DMFT estimates and the estimates themselves, thus providing a relative measure of error. The criterion used to evaluate the coefficient of variation was: values below 20% indicated estimates at acceptable levels of precision; between 20% and 30% at intermediate levels of precision, which in this study, are referred to as partially reliable; and above 30% at unacceptable levels.^17-21^

The disclosure of confidence intervals is common practice in survey result reports, allowing readers to better understand the margin of error surrounding a specific estimate. In this study, sampling errors were presented, which correspond to the semi-amplitude of these intervals. They are expressed in the unit “number of teeth” for clarity in the area, and the classes considered were less than one, between one and two, and greater than two.

The estimates of the design effect (*deff*) were also presented, corresponding to the increase imposed on the variance of dmft/DMFT due to using a complex sampling design. It is defined by the ratio of two variance estimates: the one obtained under the design actually applied and the one from the simple random sample of the same size.^22^ The considered classes were: less than or equal to two, between two and three (inclusive), between three and five (inclusive), between five and ten (inclusive), and greater than ten.

## Results

For all domains established in SB Brasil 2023, the data related to the fieldwork performed (actual sample sizes and number of surveyed census tracts), the estimates obtained for dmft/DMFT, and those regarding the evaluation of results (sampling error, coefficient of variation, and *deff*) are presented in Tables 1 [Table t2] [Table t3] [Table t4] to 5 for the ages of 5 and 12 years and the age groups of 15 to 19, 35 to 44, and 65 to 74 years, respectively.

**Table 1:** Number of interviews (n) and surveyed census tracts, dmft estimate and respective sampling error (d), coefficient of variation (cv), and design effect (*deff*) for 5-year-old children in the state capitals and Federative Units. SBBrasil 2023.

Variables	n	Census tracts	dmft	d	cv	deff
Capital						
Rio Branco	68	19	3.02	0.83	14.08	1.03
Manaus	250	29	2.02	0.48	12.20	1.76
Macapá	251	24	2.53	0.80	16.07	4.59
Belém	186	25	2.12	0.33	7.99	0.70
Porto Velho	108	23	2.37	0.72	15.38	1.45
Boa Vista	250	23	3.51	0.63	9.16	1.83
Palmas	103	42	2.29	0.75	16.62	1.22
Maceió	287	26	1.77	0.37	10.57	1.35
Salvador	292	48	1.45	0.33	11.46	1.38
Fortaleza	140	38	1.25	0.30	12.45	0.66
São Luís	134	19	1.35	0.51	19.11	1.32
João Pessoa	309	24	2.79	0.89	16.33	5.04
Recife	231	28	2.70	0.34	6.37	0.51
Teresina	111	27	1.41	0.39	14.17	0.82
Natal	204	24	2.86	0.55	9.85	1.28
Aracaju	245	32	1.79	0.30	8.48	0.82
Vitória	57	9	1.29	0.46	18.28	0.41
Belo Horizonte	132	43	1.56	0.71	23.29	1.96
Rio de Janeiro	162	26	1.59	0.66	21.20	1.83
São Paulo	275	47	1.83	0.43	11.96	1.33
Curitiba	246	48	1.59	0.52	16.60	2.28
Porto Alegre	70	26	2.15	0.77	18.30	1.31
Florianópolis	291	39	1.46	0.39	13.56	1.79
Goiânia	138	30	1.65	0.44	13.51	0.69
Campo Grande	198	39	2.17	0.59	13.77	1.31
Cuiabá	327	32	2.09	0.70	17.17	1.87
Federative unit						
AC	125	26	3.58	1.58	22.50	4.90
AM	373	40	3.06	0.71	11.80	3.74
AP	352	33	2.58	0.67	13.34	3.99
PA	287	37	2.40	0.67	14.28	4.06
RO	189	36	3.06	0.89	14.86	3.30
RR	287	27	3.70	1.00	13.77	4.50
TO	158	56	3.04	1.43	23.92	6.48
AL	352	37	2.58	1.26	24.87	12.89
BA	357	66	2.11	0.63	15.14	3.52
CE	229	53	2.13	0.62	14.89	2.50
MA	237	30	2.41	1.03	21.79	7.44
PB	375	43	1.84	0.47	13.04	2.74
PE	319	41	3.06	0.95	15.87	5.60
PI	217	43	3.05	0.83	13.93	3.47
RN	228	31	2.72	1.05	19.70	5.13
SE	324	47	2.41	0.42	8.79	1.58
ES	98	26	2.40	1.02	21.66	2.00
MG	215	66	2.03	0.78	19.60	3.24
RJ	195	43	1.73	0.69	20.26	2.36
SP	335	61	1.41	0.62	22.28	4.34
PR	327	65	1.60	0.70	22.41	5.54
RS	144	44	2.48	0.76	15.72	2.10
SC	389	57	1.46	0.57	19.83	4.39
DF	156	36	1.73	0.39	11.58	0.69
GO	218	41	3.91	1.59	20.76	10.42
MS	285	55	1.95	0.79	20.79	4.82
MT	427	52	3.04	0.55	9.24	2.57

**Table 2 t2:** Number of interviews (n) and surveyed census tracts, dmft estimate and respective sampling error (d), coefficient of variation (cv), and design effect (*deff*) for 12-year-old children in the state capitals and Federative Units. SBBrasil 2023.

Variables	n	Census tracts	dmft	d	cv	deff
Capital						
Rio Branco	61	17	2.72	0.85	15.87	1.34
Manaus	252	26	0.91	0.19	10.53	1.02
Macapá	257	28	3.45	1.06	15.64	7.26
Belém	106	18	1.68	0.47	14.38	1.53
Porto Velho	64	23	2.03	0.59	14.73	0.83
Boa Vista	250	23	4.11	0.90	11.16	2.39
Palmas	88	39	1.68	0.89	27.16	2.01
Maceió	281	26	1.61	0.21	6.65	0.78
Salvador	324	48	0.74	0.17	11.85	1.52
Fortaleza	151	43	0.70	0.19	13.93	0.79
São Luís	115	20	1.00	0.33	16.89	1.16
João Pessoa	242	37	3.28	0.80	12.40	4.69
Recife	258	28	1.49	0.52	17.74	2.67
Teresina	75	25	0.41	0.23	28.02	1.37
Natal	204	28	1.51	0.25	8.54	0.86
Aracaju	199	29	1.25	0.46	18.61	3.23
Vitória	82	19	0.68	0.25	18.71	0.92
Belo Horizonte	115	37	0.94	0.52	28.04	2.44
**Rio de Janeiro**	**123**	**24**	**1.06**	**0.90**	**43.58**	**2.60**
São Paulo	149	37	1.38	0.48	17.71	1.29
Curitiba	256	53	0.86	0.29	16.93	1.91
Porto Alegre	61	24	0.79	0.33	21.46	1.07
Florianópolis	263	37	0.77	0.30	19.82	3.00
Goiânia	131	27	1.08	0.18	8.39	1.45
Campo Grande	203	42	0.96	0.26	13.90	0.32
Cuiabá	295	31	1.75	0.81	23.72	1.66
Federative unit						
AC	118	24	3.45	1.13	16.71	3.58
AM	374	37	2.38	0.71	15.32	4.20
AP	358	38	3.10	0.90	14.87	7.22
PA	207	30	2.79	0.74	13.58	3.10
RO	148	37	2.29	0.76	17.03	2.63
RR	282	27	3.83	0.92	12.30	3.98
TO	140	51	2.67	0.84	16.06	3.07
AL	337	37	1.81	0.78	22.06	8.97
BA	403	65	1.32	0.53	20.65	7.53
CE	237	60	1.46	0.72	25.16	5.39
MA	219	31	1.82	0.78	22.01	6.32
PB	317	51	2.30	0.91	20.12	7.50
PE	341	41	2.25	1.20	27.21	22.17
PI	150	38	2.12	0.87	20.91	3.68
RN	238	37	3.07	1.14	18.98	8.71
SE	280	45	2.00	0.40	10.07	2.12
ES	130	34	1.76	0.79	22.84	1.96
MG	201	60	1.47	0.77	26.67	6.57
RJ	164	41	1.31	0.49	18.98	1.62
SP	191	48	1.08	0.45	21.36	2.97
PR	358	68	0.91	0.28	15.46	3.35
RS	138	42	1.27	0.42	16.87	2.10
SC	367	54	0.77	0.30	19.73	4.52
DF	116	29	0.73	0.33	22.84	1.45
**GO**	**206**	**37**	**3.38**	**2.09**	**31.52**	**13.68**
MS	289	58	1.72	0.54	15.96	5.57
MT	395	50	4.00	1.23	15.68	9.61

*The estimates for the bolded domains have unacceptable precision according to the cv.

**Table 3 t3:** Number of interviews (n) and surveyed census tracts, dmft estimate and respective sampling error (d), coefficient of variation (cv), and design effect (*deff*) for adolescents aged 15 to 19 years in the state capitals and Federative Units. SBBrasil 2023.

Variables	n	Census tracts	DMFT	d	cv	deff
Capital						
Rio Branco	129	19	4.1	1.0	12.3	2.02
Manaus	313	32	2.6	0.7	13.7	4.30
Macapá	301	25	3.9	0.7	9.4	2.89
Belém	165	25	3.1	0.9	14.7	2.15
Porto Velho	115	26	4.2	0.8	9.9	1.01
Boa Vista	299	23	6.2	1.2	9.6	3.19
**Palmas**	**110**	**37**	**4.5**	**2.9**	**32.8**	**4.59**
Maceió	304	27	4.5	2.5	28.5	8.57
Salvador	331	48	1.6	0.6	20.2	2.33
Fortaleza	202	40	2.0	0.5	13.3	1.64
São Luís	195	25	3.0	0.5	8.5	0.93
João Pessoa	292	41	4.0	1.1	14.1	4.79
Recife	302	33	3.2	0.7	11.5	1.65
Teresina	121	32	1.8	0.5	14.4	0.73
Natal	259	28	2.9	0.5	9.7	1.64
Aracaju	218	33	3.0	0.8	14.0	1.53
Vitória	74	23	1.9	0.9	23.1	2.09
Belo Horizonte	148	45	2.0	0.8	21.2	1.45
Rio de Janeiro	196	28	2.4	1.3	27.7	3.31
São Paulo	201	43	3.7	0.9	11.8	1.70
Curitiba	294	48	2.4	0.5	11.2	1.11
Porto Alegre	113	32	1.4	0.6	21.0	1.84
Florianópolis	338	48	2.1	0.8	18.1	2.37
Goiânia	132	25	2.9	1.0	17.6	1.34
Campo Grande	281	41	3.1	0.4	7.2	2.05
Cuiabá	304	30	3.9	1.5	19.3	1.25
Federative unit						
AC	191	26	5.7	2.0	17.7	7.39
AM	405	43	3.7	0.9	12.3	5.25
AP	401	35	4.4	1.1	12.6	4.34
PA	274	36	5.1	2.1	20.6	12.73
RO	213	38	4.7	1.4	14.6	4.32
RR	337	27	6.0	1.0	8.1	2.71
TO	185	49	5.7	1.0	9.0	1.61
AL	429	39	4.4	1.8	21.1	10.79
BA	406	64	2.8	0.7	12.9	3.06
CE	281	55	4.1	1.7	20.8	9.31
MA	248	35	3.3	1.5	22.9	5.05
PB	356	59	3.8	1.0	13.8	6.16
PE	362	46	3.6	1.0	13.8	6.35
PI	183	46	4.1	1.4	17.5	5.01
RN	309	38	5.1	2.1	20.7	13.19
SE	286	48	2.9	0.6	10.9	1.82
ES	122	35	3.3	0.9	13.7	2.09
MG	254	69	3.0	0.7	11.8	2.67
RJ	276	49	2.7	0.9	16.5	2.90
SP	267	56	2.8	1.1	20.8	5.68
PR	403	64	2.9	1.3	22.2	11.68
RS	219	52	2.3	1.1	24.3	3.99
SC	466	68	1.5	0.4	13.1	3.35
DF	212	42	2.0	0.5	13.9	1.34
GO	199	35	6.0	1.9	15.8	5.34
MS	364	57	4.1	0.9	10.9	3.54
MT	406	48	7.3	2.5	17.4	20.29

*The estimates for the bolded domains have unacceptable precision according to the cv.

**Table 4 t4:** Number of interviews (n) and surveyed census tracts, dmft estimate, and respective sampling error (d), coefficient of variation (cv), and design effect (*deff*) for participants aged 35 to 44 years. in the state capitals and Federative Units. SBBrasil 2023.

Variables	n	Census tracts	DMFT	d	cv	deff
Capital						
Rio Branco	136	20	11.27	2.5	11.46	3.76
Manaus	312	32	11.03	0.9	4.19	1.73
Macapá	359	17	7.88	1.4	9.01	4.87
Belém	262	24	7.60	1.5	10.36	3.69
Porto Velho	151	28	12.81	1.5	5.86	2.09
Boa Vista	304	27	10.87	1.7	7.87	3.90
Palmas	147	40	10.62	2.2	10.48	3.18
Maceió	322	25	11.38	1.3	5.86	3.70
Salvador	325	47	10.27	0.6	3.15	0.89
Fortaleza	245	41	10.88	1.0	4.72	1.87
São Luís	267	29	9.35	0.9	5.04	1.84
João Pessoa	266	46	12.12	1.8	7.66	4.51
Recife	293	34	12.96	1.9	7.31	3.17
Teresina	147	36	10.39	1.3	6.40	1.63
Natal	240	33	12.93	1.0	3.90	1.27
Aracaju	311	36	8.17	1.2	7.29	2.38
Vitória	112	27	8.66	1.8	10.55	2.19
Belo Horizonte	221	42	7.91	1.0	6.57	1.65
Rio de Janeiro	253	38	8.94	1.5	8.72	3.77
São Paulo	268	41	12.00	1.1	4.61	2.01
Curitiba	306	48	10.16	1.3	6.69	2.91
Porto Alegre	210	40	7.25	1.0	6.69	1.42
Florianópolis	298	50	8.38	1.3	8.11	2.55
Goiânia	177	27	11.24	1.8	8.22	1.59
Campo Grande	275	38	10.59	1.1	5.46	3.34
Cuiabá	340	28	10.60	2.1	10.34	2.39
Federative unit						
AC	192	28	12.6	2.5	10.0	5.14
AM	417	43	11.9	1.2	5.2	3.43
AP	461	25	8.2	2.2	13.7	11.93
PA	363	36	10.5	3.2	15.5	16.86
RO	261	40	13.4	1.9	7.1	5.00
RR	346	31	11.4	2.0	9.0	7.04
TO	213	52	11.9	1.6	6.8	2.91
AL	445	37	11.0	2.2	10.1	12.54
BA	400	64	11.0	2.4	11.1	11.52
CE	344	55	11.8	2.6	11.2	10.94
MA	325	41	7.1	3.0	21.8	22.18
PB	361	65	11.5	1.8	8.2	6.57
PE	373	48	11.2	1.7	7.8	5.41
PI	217	50	9.3	2.8	15.5	9.36
RN	286	42	10.5	2.8	13.5	13.32
SE	373	49	8.7	1.7	9.7	6.82
ES	162	45	8.7	1.5	8.8	2.64
MG	322	66	10.9	1.9	8.9	5.77
RJ	330	60	10.0	1.3	6.5	3.84
SP	341	56	11.9	1.6	6.8	5.27
PR	423	66	9.4	1.4	7.5	5.41
RS	297	58	8.1	1.0	6.2	2.43
SC	420	68	9.4	2.1	11.6	11.32
DF	243	43	9.3	0.9	5.0	1.59
GO	287	37	11.9	1.6	6.8	3.34
MS	377	54	10.5	1.1	5.4	2.92
MT	440	43	12.9	2.9	11.4	15.91

*The estimates for the bolded domains have unacceptable precision according to the cv.

**Table 5:** Number of interviews (n) and census tracts surveyed, dmft estimate and respective sampling error (d), coefficient of variation (cv), and design effect (*deff*) for participants aged 65 to 74 years. in the state capitals and Federative Units. SBBrasil 2023.

Variables	n	Census tracts	DMFT	d	cv	deff
Capital						
Rio Branco	157	21	23.9	2.5	5.4	2.86
Manaus	299	33	26.0	0.7	1.4	1.01
Macapá	303	18	22.6	3.3	7.5	8.28
Belém	185	28	23.0	1.8	4.0	2.14
Porto Velho	144	28	23.4	1.9	4.2	1.89
Boa Vista	304	23	25.3	1.8	3.7	2.81
Palmas	166	44	25.1	1.6	3.2	1.30
Maceió	328	28	23.3	1.7	3.7	3.03
Salvador	319	47	21.2	1.0	2.4	1.55
Fortaleza	293	44	25.6	1.1	2.2	1.88
São Luís	222	28	24.9	1.2	2.4	1.43
João Pessoa	270	43	24.8	1.9	3.9	3.54
Recife	273	35	24.3	1.6	3.4	2.47
Teresina	196	35	23.9	1.4	3.0	2.03
Natal	272	32	26.1	1.4	2.7	2.76
Aracaju	228	34	20.9	2.3	5.7	2.97
Vitória	82	22	17.3	1.9	5.6	0.93
Belo Horizonte	241	48	23.4	2.1	4.6	3.68
Rio de Janeiro	388	41	22.2	1.8	4.2	3.65
São Paulo	290	48	24.1	1.6	3.4	2.57
Curitiba	302	56	22.4	1.4	3.3	2.02
Porto Alegre	270	45	18.5	1.7	4.8	2.33
Florianópolis	321	50	20.3	1.6	4.1	2.41
Goiânia	204	31	23.3	1.2	2.7	1.48
Campo Grande	323	42	22.3	1.5	3.3	1.34
Cuiabá	306	29	26.4	1.8	3.4	1.97
Federative unit						
AC	218	29	4.1	1.0	12.3	2.40
AM	406	44	2.6	0.7	13.7	2.61
AP	404	28	3.9	0.7	9.4	6.65
PA	326	39	3.1	0.9	14.7	8.48
RO	297	42	4.2	0.8	9.9	1.95
RR	336	27	6.2	1.2	9.6	1.62
**TO**	**256**	**58**	**4.5**	**2.9**	**32.8**	**4.84**
AL	450	40	4.5	2.5	28.5	4.76
BA	423	64	1.6	0.6	20.2	9.99
CE	400	61	2.0	0.5	13.3	5.42
MA	302	39	3.0	0.5	8.5	7.91
PB	362	60	4.0	1.1	14.1	7.05
PE	399	49	3.2	0.7	11.5	3.86
PI	293	50	1.8	0.5	14.4	2.65
RN	338	42	2.9	0.5	9.7	7.12
SE	304	49	3.0	0.8	14.0	10.24
ES	169	41	1.9	0.9	23.1	1.80
MG	355	74	2.0	0.8	21.2	4.03
RJ	519	63	2.4	1.3	27.7	5.02
SP	415	64	3.7	0.9	11.8	5.44
PR	507	74	2.4	0.5	11.2	2.44
RS	446	68	1.4	0.6	21.0	4.26
SC	472	71	2.1	0.8	18.1	6.47
DF	244	43	2.9	1.0	17.6	1.48
GO	281	42	3.1	0.4	7.2	5.43
MS	417	59	3.9	1.5	19.3	3.65
MT	406	46	4.1	1.0	12.3	7.98

*The estimates for the bolded domains have unacceptable precision according to the cv.

Most estimates of dmtf/DMTF (80%) were precise, both for the capitals (88.5%) and for the Federative Units (71.8%). The index age of 12 years had the lowest percentage of precise samples (66.0%). Four estimates (1.5%) had unacceptable precision levels when considering the coefficient of variation criterion. It is also observed that the estimates were more precise for the capitals than for the Federative Units at all ages (Table 6).

**Table 6:** Number and percentage of samples by geographical and demographic domains, according to the coefficient of variation (%) of the dmft and DMFT estimates.

Demographic (Age)	Coefficient of variation (%)							
	< 20		20–30		> 30		Total	
	n	%	n	%	n	%	n	%
Capital								
5	24	92.3	2	7.37	-	-	26	100.0
12	20	76.9	5	19.2	1	3.8	26	100.00
15–19	19	73.1	6	23.1	1	3.8	26	100.0
35–44	26	100.0	-	-	-	-	26	100.0
65–74	26	100.0	-	-	-	-	26	100.0
Total	115	88.5	13	10.0	2	1.5	130	100.0
Federative unit								
5	17	63.0	10	37.0	-	-	27	100.0
12	15	55.6	11	40.7	1	3.7	27	100.0
15–19	19	70.4	8	29.6	-	-	27	100.0
35–44	26	96.3	1	3.7	-	-	27	100.0
65–74	20	74.1	6	22.2	1	3.7	27	100.0
Total	97	71.8	36	26.7	2	1.5	135	100.0
Capital + Federative unit								
5	41	77.4	12	22.6	-	-	53	100.0
12	35	66.0	16	30.2	2	3.8	53	100.0
15–19	38	71.7	14	26.4	1	1.9	53	100.0
35–44	52	98.1	1	1.9	-	-	53	100.0
65–74	46	86.8	6	11.3	1	1.9	53	100.0
Total	212	80.0	49	18.5	4	1.5	265	100.0

Regarding sampling error, the absolute values observed for most estimates (59.6%) were below one, equivalent to a margin of error of one tooth for the confidence intervals. As expected, DMFT estimates rise with increasing age, and consequently, the absolute error value increases. However, even in adult and elderly groups, this error exceeded two in less than 20% of the samples. Estimates for Federative Units had higher absolute error values than those for the capitals, indicating lower precision levels, as also noted by the coefficients of variation (Table 7).

**Table 7:** Number and percentage of samples by geographical and demographic domains, according to sampling errors of dmft and DMFT estimates.

Demographic (Age)	Sampling Error							
	< 1		1–2		> 2		Total	
	n	%	n	%	n	%	n	%
Capital								
5	26	100.00	-	-	-	-	26	100.0
12	25	96.2	1	3.8	-	-	26	100.0
15–19	20	76.9	4	15.4	2	7.7	26	100.0
35–44	7	26.9	16	61.5	3	11.6	26	100.0
65–74	2	7.7	20	76.9	4	15.4	26	100.0
Total	80	61.5	41	31.5	9	6.9	130	100.0
Federative unit								
5	20	74.1	7	25.9	-	-	27	100.0
12	22	81.5	4	14.8	1	3.7	27	100.0
15–19	13	48.1	11	40.7	3	11.2	27	100.0
35–44	2	7.4	14	51.9	11	40.7	27	100.0
65–74	21	77.8	4	14.8	2	7.4	27	100.0
Total	55	57.8	55	29.6	25	12.6	135	100.0
Capital + Federative unit								
5	46	86.8	7	13.2	-	-	53	100.0
12	47	88.7	5	9.4	1	1.9	53	100.0
15–19	33	62.3	15	28.3	5	9.4	53	100.0
35–44	9	17.0	30	56.6	14	26.4	53	100.0
65–74	23	43.4	24	45.3	6	11.3	53	100.0
Total	158	59.6	81	30.6	26	9.8	265	100.0

Regarding the *deff*, the differences between the samples from the capitals and the Federative Units were pronounced. For the capitals, more than half (55.4%) of the samples had *deff* lesser than two, which occurs in only 10.4% of the samples from the Federative Units. At the opposite extreme, no sample from the capitals has *deff* estimates larger than 10, while in the Federative Units, 14.1% are at this level (Table 8).

**Table 8:** Number and percentage of samples by geographical domains, according to the design effect (*deff*) cut points of the dmft and DMFT estimates.

deff	Capital		Federative unit	
	n	%	n	%
≤ 2	72	55.4	14	10.4
2–3	32	24.6	21	15.6
3–5	22	16.9	36	26.7
5–10	4	3.1	45	33.3
> 10	0	0.0	19	14.1
total	130	100	135	100

## Discussion

The precision of dmft/DMFT estimates was adequate for most study domains. However, when considering only the Federative Units-level samples, the proportion of results classified as partially reliable cannot be regarded as negligible, as one-fifth of them fall into this category.

The dmft/DMFT estimates for the Federative Units showed lower precision across all age groups compared to capitals. This suggests that the sample sizes allocated to non-capital areas (the interior), which should have been added to those in the capitals, were insufficient. However, it is important to note that for all estimates classified as partially reliable, the sample sizes for the interior were below the 100 interviews proposed in the sampling plan. Additionally, differences in the base weights between interior and capital census tracts, combined with variations in oral health indicators between these areas may have negatively impacted the variance estimates. Large differences in the number of interviews per census tracts, an undesirable aspect in sampling plans^23^, may have also occurred due to issues in the field, including registration problems, a hypothesis still under investigation.

Although no precision criteria were applied to the absolute values of the sampling errors, knowing the semi-amplitude of confidence intervals (the distance between the interval limits and the point estimate) helps oral health professionals better understand the precision of the estimates. Most dmft/DMFT estimates had errors below one for the younger age groups and below two for the older groups, values that reflect the previous information that most of the estimates were precise. These results are also useful for calculating sample sizes in future sampling plans.

The *deff*, also evaluated in this study, has been considered an extremely useful tool in developing efficient sampling plans^23,24^. Several aspects of complex plans impact the *deff*, including the clustering of elements into sampling units, which gives rise to intraclass correlation, and the selection of units with unequal probabilities, which leads to the use of weights in the data analysis stage. Intraclass correlation is a characteristic present in the population, and to minimize its impact on variance estimates, efforts are made to select a small number of units in each cluster. In this study, the average number of people interviewed in each census tract was less than 15, except for three samples, a number considered adequate for this average.^24^ Regarding the weights, the goal is to control their variability.^23,24^

The *deff* values indicated higher efficiency of the samples in the capitals compared to the Federative Units. According to Kish, the efficiency of a sampling design refers to the fulfillment of the research objectives expressed in terms of precision, under a fixed minimum cost.^22^ In this sense, *deff* can be considered an efficiency indicator, as it can be seen as the increase in the size of a simple random sample needed for the estimates to have the desired precision when obtained through complex sampling.

There are aspects of the fieldwork that must be considered in the analysis of the precision of the results and the sample efficiency. As mentioned in the description of the sampling plan, difficulties encountered during the fieldwork led to the introduction of several adjustments in the sampling fractions applied. As a result, census tracts within the same stratum had very distinct base weights, which may have contributed to an increase in the variability of the weights and, consequently, the *deff* ^2^ Among the difficulties mentioned, a high non-response rate of households stands out,^8^ resulting in insufficient number of eligible residents being identified for the survey and changes in the drawing fractions to approach the desired sample size. The early abandonment of the household listing activity in some census tracts also led to the inclusion of reserve census tracts and households, previously drawn.

The introduction of changes in sampling fractions also resulted from using highly outdated data in the sample planning. The person-to-household ratios, which guide the determination of the number of households to be visited, were established based on data obtained a long time before the fieldwork.^6^ As a result, the number of eligible people for the survey found was far from what was expected. Additionally, drawing a fixed number of households in census tracts with current sizes different from those used in the planning caused the sample to not benefit from the self-weighting intended in the drawing with probability proportional to size.

Regarding factors involved in the variation of weights, the drawing of census tracts with probability proportional to size is also mentioned, leading to the inclusion of the entire eligible population in the sample. This occurred for children and adolescents aged 5 to 12 years, as the same census tracts were used for all age groups. Furthermore, the introduction of adjustments to align the distribution of sociodemographic variables in the sample with the distribution of these variables in the population, aiming to reduce potential biases from non-response, may also have increased the dispersion of the weights.^23^

## Conclusion

Most of the estimates were precise, both for the capitals and for the Federative Units, with the estimates for the capitals showing smaller errors than those for the Federative Units. Additionally, the efficiency indicator showed that the estimates for the capitals were superior to those for the Federative Units.

Increasing the levels of precision in the estimates and efficiency of the samples in the Federative Units remains a challenge to be addressed in future editions of the survey. Despite the limitations mentioned, the adoption of this unit of analysis in the SB Brasil 2023 represented a significant advancement, addressing a strategic demand of public management. For the first time in the country, efforts were made to estimate specific indicators for each Federative Unit through a national survey, enhancing the capacity for planning and evaluating state policies. Obtaining data for the Federative Units represents an important milestone, providing support to improve future research and strengthening oral health surveillance in the country.
